# Development of a Smart Hallway for Marker-Less Human Foot Tracking and Stride Analysis

**DOI:** 10.1109/JTEHM.2021.3069353

**Published:** 2021-03-29

**Authors:** Vinod Gutta, Pascal Fallavollita, Natalie Baddour, Edward D. Lemaire

**Affiliations:** 1 School of Electrical Engineering and Computer ScienceUniversity of Ottawa 6363 OttawaONK1N 6N5Canada; 2 Interdisciplinary School of Health SciencesUniversity of Ottawa 6363 OttawaONK1N 7K4Canada; 3 Department of Mechanical EngineeringUniversity of Ottawa 6363 OttawaONK1N 6N5Canada; 4 The Ottawa Hospital Research Institute 10055 OttawaONK1H 8M2Canada; 5 Faculty of MedicineUniversity of Ottawa 6363 OttawaONK1H 8M5Canada

**Keywords:** Foot tracking, Intel RealSense D415, marker-less, smart hallway, stride analysis

## Abstract

Objective: In this research, a marker-less ‘smart hallway’ is proposed where stride parameters are computed as a person walks through an institutional hallway. Stride analysis is a viable tool for identifying mobility changes, classifying abnormal gait, estimating fall risk, monitoring progression of rehabilitation programs, and indicating progression of nervous system related disorders. Methods: Smart hallway was build using multiple Intel RealSense D415 depth cameras. A novel algorithm was developed to track a human foot using combined point cloud data obtained from the smart hallway. A method was implemented to separate the left and right leg point cloud data, then find the average foot dimensions. Foot tracking was achieved by fitting a box with average foot dimensions to the foot, with the box’s base on the foot’s bottom plane. A smart hallway with this novel foot tracking algorithm was tested with 22 able-bodied volunteers by comparing marker-less system stride parameters with Vicon motion analysis output. Results: With smart hallway frame rate at approximately 60fps, temporal stride parameter absolute mean differences were less than 30ms. Random noise around the foot’s point cloud was observed, especially during foot strike phases. This caused errors in medial-lateral axis dependent parameters such as step width and foot angle. Anterior-posterior dependent (stride length, step length) absolute mean differences were less than 25mm. Conclusion: This novel marker-less smart hallway approach delivered promising results for stride analysis with small errors for temporal stride parameters, anterior-posterior stride parameters, and reasonable errors for medial-lateral spatial parameters.

## I. Introduction

Human stride analysis in clinical settings is often performed with optical marker tracking systems such as Vicon, Optitrack, and Qualisys, requiring expensive setup, specialized human resources, and dedicated laboratory space. Passive or active markers, such as light-emitting diodes, are placed on the body to track limbs for human gait acquisition and characterization [1]. Inertial Measurement Units (IMU) can also be attached to body parts to record inertial motion based [2] kinematic gait data. Affixing external sensors on the human body may cause discomfort to patients and substantially change their natural gait [3]. These systems also require technical expertise for attaching markers and conducting experiments.

Low-cost Kinect depth sensors for gaming showed potential for human gait-related health care applications; such as, fall risk [4]–[6], Parkinson’s disease movement assessment [7], fall detection of people with multiple sclerosis [8], autism disorder identification [9], abnormal gait classification [10], [11], virtual gait training [12], diagnosis, monitoring, and rehabilitation [13]. Depth sensors capture both depth and color images. Depth data contains distances, at each pixel, between the depth sensor and objects in the capturing scene. With this depth information, the real 3D coordinates at each pixel are recorded, with the depth sensor as the origin (i.e., “point cloud”). Most research on depth sensors for human movement analysis involved Microsoft Kinect. Kinect V2 systems can identify and track the majority of human joints by defining joint locations that constitute a human skeleton-model or building a human model from the scene’s point cloud. However, gaps and limitations exist with frame rate [14] and lower body tracking, especially at the ankle and foot [14], [15]. Multiple Kinect V2 sensors can capture longer volumes, with promising results, but ankle tracking farther from the sensor was more inconsistent [16]. Kinect’s machine learning-based skeleton tracking was not reliable, with tracking points sometimes moving outside the body and tracking varying with viewing angle [17]. Given these limitations, approaches that use the whole point cloud could provide better foot tracking results.

Patients, staff, and visitors typically move through similar hallways in hospitals, rehabilitation centers, or long-term care facilities [18]. This space could be utilized in an intelligent way by building a system to perform marker-less stride analysis as patients or residents walk through the hallway. Measuring patients every time they walk through the hallway, without intervention, could help identify changes in their movement status. To capture multiple strides and avoid occlusions while a person is walking, multiple depth sensors would be required. Unlike time-of-flight based Kinect V2 sensor, Stereoscopic infra-red based Intel RealSense D415 depth sensors were shown to be suitable for this application since they did not experience interference when multiple sensors were used simultaneously [19]. Furthermore, these sensors capture data at 60fps, which can provide sufficient data for analyzing temporal related stride parameters.

This research explored depth sensing technology for stride parameter analysis within an institutional hallway environment. The main contributions were developing, prototyping, and validating a novel point-cloud-based marker-less system with an innovative foot tracking algorithm and assessed system performance by comparing stride parameter output with industry standard marker-based motion analysis. Successful implementation of this smart hallway concept would introduce unobtrusive movement status assessment that can guide clinical decision-making, without introducing unsustainable human resource requirements. This would also become the basis for future data analytics applications for predicting changes in dementia, fall risk, or other aging-related conditions.

## II. System Setup and Synchronization

**FIGURE 1.**
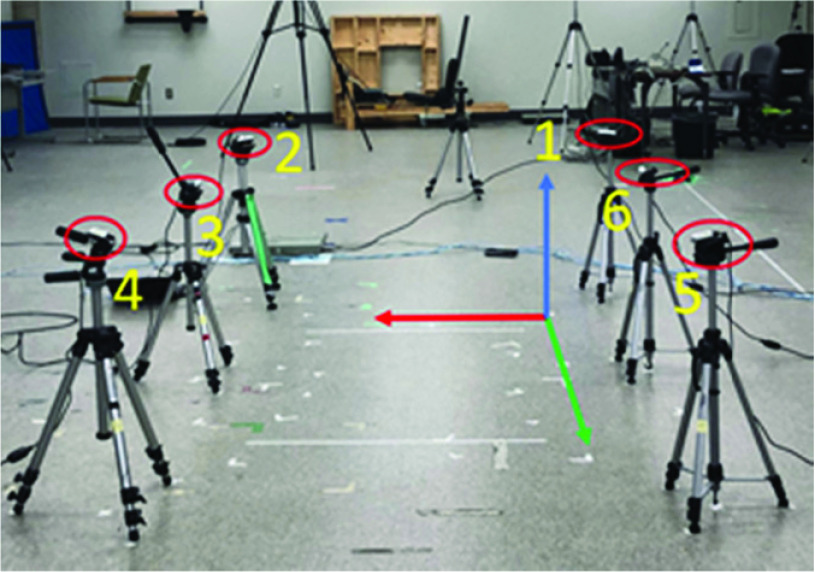
Physical setup of the marker-less system using hallway dimensions (Red: x-axis, Green: y-axis, and Blue: z-axis).

In our previous work [20], a physical setup with six Intel RealSense D415 sensors was configured in the lab to replicate a hallway scenario (Fig. 1). These sensors were placed at 0.8m height from the floor, with 1.4m between adjacent sensors in y-axis (i.e., (1,6), (2,3), (3,4), (5,6)), and 1.8m in x-axis direction (i.e., (1,2), (3,6) and (4,5)). Techniques from this research were used for temporal synchronization (client-server approach) and spatial synchronization (stereo chess board method).

The new smart hallway system captures $848\times480$ pixel depth images and color images at approximately 60fps (frame rate varies slightly because of buffer time for transmitting data through Ethernet after capturing each frame), with a timestamp for each frame, from all the six sensors. To increase accuracy and reduce computation time, a non-zero median filter for every $2\times2$ pixels was applied to remove “spikes” in the depth data and down-sample to half resolution ($424\times240$) [21].

### A. Sensor Parameters

Intrinsic parameters such as focal length ($f_{x}, f_{y}$) and principal point ($c_{x}, c_{y}) $ for Intel RealSense D415 depth and color cameras were obtained from the manufacturer. Two coordinate systems (depth, color) and extrinsic parameters (rotation and translation, to transform data between depth and color coordinate systems) were also obtained. This marker-less six depth sensors setup was spatially synchronized in the color coordinate system, such that the combined output point cloud was in the first sensor’s color coordinate system.

### B. Room Coordinate System

**FIGURE 2.**
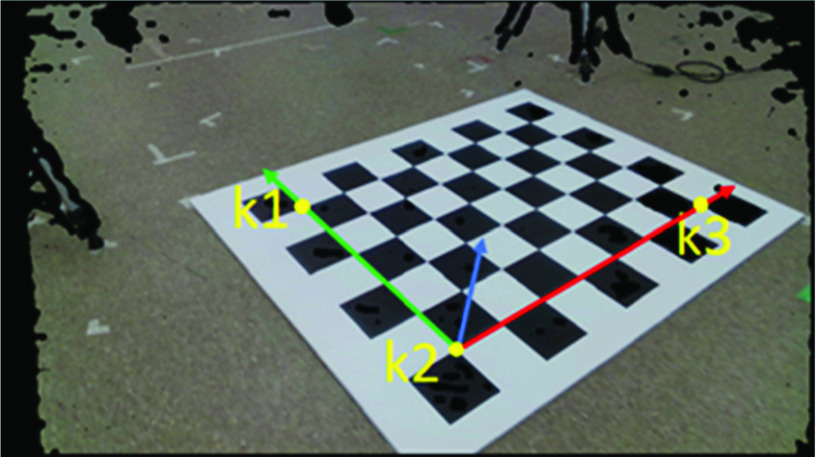
Key points on the chessboard for room coordinate system (Red: x-axis, Green: y-axis, and Blue: z-axis).

A reference coordinate system on the floor plane (Fig. 1) was designed using a chessboard (Fig. 2), with x-axis in the medial-lateral (ML) walking direction, y-axis parallel to the walking pathway (anterior-posterior; AP), and z-axis outwards to the floor (Vertical; V). This reference coordinate system was labelled as the Room Coordinate System (RCS). Methods from our previous study [20] were used to transform point cloud data from all the sensors into the first sensor’s color coordinate system, then transformed into the RCS by determining a transformation matrix $T_{R\leftarrow 1}$ (1).

A $8\times6$ chessboard was placed on the floor with the horizontal edge parallel to the RCS x-axis and vertical edge parallel to the y-axis. The board’s depth and color images were captured with the first sensor at $1280\times720$ resolution. The depth image was down-sampled to half resolution using median filter. 3D points were calculated from the depth image using depth intrinsic parameters and then the points were transformed into the color coordinate system using extrinsic parameters.

A new color image was constructed using 3D points and corresponding projection pixels onto the captured color image. For a 3D point ($x,y,z$) in the color coordinate system corresponding to row $r_{d}$ and column $c_{d}$ in the depth image, the projected pixel location ($r_{c}, c_{c}$) in the captured color image was found using equations (2) and (3) (color camera’s intrinsic parameters). The red, green, and blue channel values at row $r_{d}$ and column $c_{d}$ in the constructed color image (Fig. 2) were the values at row $r_{c}$ and column $c_{c}$ in the captured color image and the values of pixels corresponding to invalid 3D data (0,0,0) were set to zeroes.

Three key points $k_{1}, k_{2}, $ and $k_{3}$ were identified in 100 frames. For each key point, 100 instants of its 3D location were obtained in the color coordinate system. Each dimension value ($x$, $y$, $z$) of these 100 3D points was sorted separately and the middle 50 values were averaged.

The RCS origin was at point $k_{2} {(x}_{0}, \mathrm { }y_{0}, z_{0})$, the unit vectors were $\hat {x_{R}} (x_{R}^{x},{y}_{R}^{x}, \mathrm { }z_{R}^{x})$ from $k_{2}$ to $k_{3}$, $\hat {y_{R}} \mathrm { }(x_{R}^{y},{y}_{R}^{y}, z_{R}^{y})$ from $k_{2}$ to $k_{1}$, and $\hat {z_{R}} (x_{R}^{z},{\mathrm { }y}_{R}^{z}, z_{R}^{z})$ the cross product of $\hat {x_{R}}$ and $\hat {y_{R}}$ with respect to the first sensor color coordinate system, whose origin was at the point (0, 0, 0) and corresponding unit vectors were $\hat {x}(1,0,0)$, $\hat {y}\left ({0,1,0 }\right)$, and $\hat {z}\left ({0,0,1 }\right)$, respectively. The transformation matrix from the first sensor to the RCS was obtained using (1).

## III. Point Cloud

A point cloud was generated from the six depth sensors. The process involved generating a background depth image from a static scene, subtracting the background information from the depth images, constructing walking human point cloud data for each sensor from the background subtracted depth images, and merging and transforming point clouds from the six sensors to RCS. The combined point cloud was filtered and smoothed to reduce noise.

### A. Background Frame

From each sensor, 1000 depth frames of background data (without any objects) were captured. The pixel value at row $y$, column $x$ of these background frames was represented as ${bf}_{yx}^{(j, i)}$ for the $i^{th}$ frame of the $j^{th}$ sensor. This system was designed to work in the range of 200mm to 5000mm. All background frame pixels for the $j^{th}$ sensor $\left ({{BF}^{\left ({j }\right)} }\right)$ were initialized with 5000 (4), then the pixel value at row $y$, column $x {(BF}_{yx}^{(j)})$ was updated with the minimum of ${BF}_{yx}^{(j)} $ and ${bf}_{yx}^{(i)}$, iterating through 1000 frames ($i=1$ to 1000) using eq. (5).

### B. Background Subtraction

A background subtracted depth image for the $j^{th}$ sensor $({BS}^{\left ({j }\right)})$ was obtained by pixel-wise comparison with the corresponding sensor’s background frame ${(BF}^{(j)})$ [22]. For a depth frame from the $j^{th} \mathrm { }$ sensor $({DF}^{\left ({j }\right)})$, pixel values less than the background frame’s pixel value, and greater than the minimum value (200 mm), were considered the same value in the ${BS}^{\left ({j }\right)}$ frame. Other pixel values were assigned the maximum value (5000 mm), as presented in eq. (6), for a pixel in $y^{th}$ row and $x^{th}$ column. For further processing, ${BS}^{\left ({j }\right)}$ was linearly scaled down to [0, 255] from [0, 5000].

\begin{align*}T_{R\leftarrow 1}=&\left [{ {\begin{array}{cccccccccccccccccccc} x_{R}^{x} &\quad x_{R}^{y} &\quad x_{R}^{z} &\quad x_{0}\\ y_{R}^{x} &\quad y_{R}^{y} &\quad y_{R}^{z} &\quad y_{0}\\ z_{R}^{x} &\quad z_{R}^{y} &\quad z_{R}^{z} &\quad z_{0}\\ 0 &\quad 0 &\quad 0 &\quad 1\\ \end{array}} }\right]^{-1} \tag{1}\\ r_{c}=&\left \lfloor{ \left ({\frac {y\times f_{y}}{z} }\right)+c_{y} }\right \rfloor \tag{2}\\ c_{c}=&\left \lfloor{ \left ({\frac {x\times f_{x}}{z} }\right)+c_{x} }\right \rfloor\tag{3}\end{align*}

From the scaled-down image ${(SBS}^{\left ({j }\right)})$, a Binary Background Subtracted image $({BBS}^{\left ({j }\right)})$ was constructed based on eq. (7).

**FIGURE 3.**
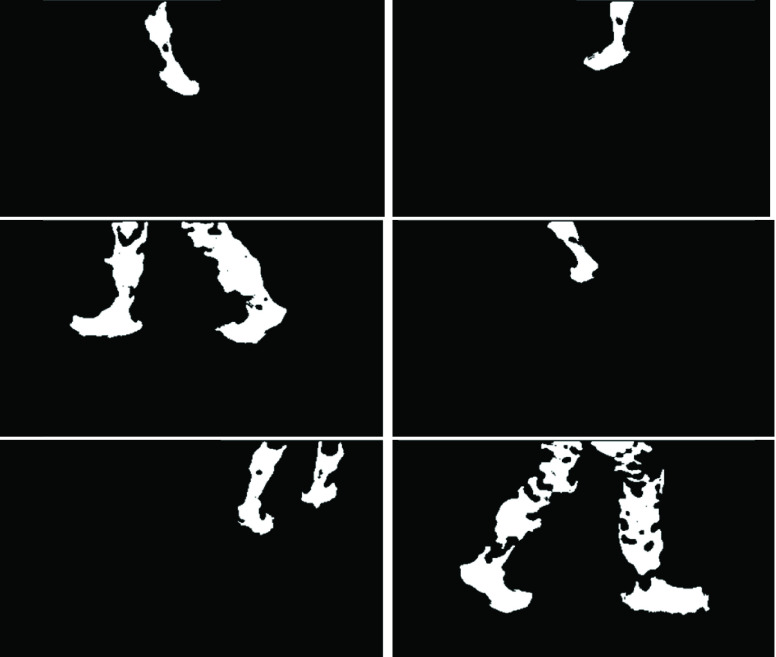
Background subtracted binary images from six sensors.

A connected component filter [23] with 1000 pixels connected area cut-off was applied to the ${BBS}^{\left ({j }\right)}$ image and output was a Binary Filtered Background Subtracted image $({BFBS}^{\left ({j }\right)})$. The ${BS}^{(j)}$ image was modified based on the ${BFBS}^{\left ({j }\right)}$, pixel locations with zero value in ${BFBS}^{\left ({j }\right)}$ were assigned to zero in ${BS}^{(j)}$ frame (8). Sample $BFBS$ frames from all sensors are shown in Fig. 3. White pixels in the $BFBS$ frames were foreground and black pixels were background. Depth data was not captured in the small gaps among foreground pixels.

### C. Point Cloud Construction

3D point cloud points were constructed from each sensor’s background-subtracted depth images and then transformed into the first sensor’s coordinate system [20]. This “combined point cloud” points were transformed into RCS by multiplying with transformation matrix $T_{R\leftarrow 1}$, obtained from (1).

\begin{align*}&\hspace {-2pc}{BF}_{yx}^{(j)} \\=&5000;~1\le y \le 424,~ 1\le x \le 240 \tag{4}\\&\hspace {-2pc}{BF}_{yx}^{(j)} \\=&\begin{cases} {BF}_{yx}^{(j)};& { bf}_{yx}^{(j, i)} < 200 or {bf}_{yx}^{(j, i)} > 5000 \\ {BF}_{yx}^{\left ({j }\right)};&200 < {bf}_{yx}^{\left ({j, i }\right)} < 5000, {BF}_{yx}^{(j)} {\le bf}_{yx}^{(j, i)} \\ {bf}_{yx}^{\left ({j }\right)};& 200 < {bf}_{yx}^{\left ({j, i }\right)} < 5000, {BF}_{yx}^{(j)} { > bf}_{yx}^{(j, i)} \\ \end{cases}\tag{5}\\&\hspace {-2pc}{BS}^{(j)}_{yx_{y\in \left \{{1,2, \ldots,424 }\right \}, x\in \{1,2, \ldots, 240\}}} \\=&\begin{cases} {DF}_{yx}^{(j)}; & {DF}_{yx}^{(j)} < {BF}_{yx}^{(j)}, {DF}_{yx}^{(j)} > 200\\ 5000; & Other wise\\ \end{cases}\tag{6}\\&\hspace {-2pc}{BBS}^{(j)}_{yx_{y\in \left \{{1,2, \ldots,424 }\right \}, x\in \{1,2, \ldots, 240\}}} \\=&\begin{cases} 0; & {SBS}_{yx}^{(j)}\ge 250\\ 255; & {SBS}_{yx}^{(j)} < 250\\ \end{cases}\tag{7}\\&\hspace {-2pc} {BS}^{(j)}_{yx_{y\in \left \{{1,2, \ldots,424 }\right \}, x\in \{1,2, \ldots, 240\}}} \\=&\begin{cases} 0; & {BFBS}_{yx}^{(j)}=0\\ {BS}_{yx}^{(j)}; & {BFBS}_{yx}^{(j)}=255\\ \end{cases}\tag{8}\end{align*}

### D. Point Cloud Filtering

The combined point cloud was filtered using a statistical outlier filter [24], smoothed with a moving least-squares technique [25], and then down-sampled with a voxel grid filter [26]. OpenCV libraries [27] were used for 2D image processing and PCL [28] for 3D point cloud processing.

For every 3D point in a point cloud, 100 neighbor points were analyzed to find outliers. Mean and standard deviation of distances of the closest 100 points from each point of interest were found. Points farther than one standard deviation from the point of interest were considered outliers and removed.

Point cloud points were smoothed by fitting a second-order polynomial equation to points within 30 mm of each point of interest in the point cloud. The point cloud was divided into 5mm $\times5$mm $\times5$mm voxels (3D boxes) and then down-sampled by replacing points in a voxel with the centroid of these points. This method of down-sampling retained the point cloud surface and reduced computation time for point cloud processing.

## IV. Leg Segmentation

Since this application tracks a walking person’s foot, point cloud points less than 70 cm from the floor were selected, since the foot and shank are always present in this region. The free parameters presented in the following sections were tuned to fit the foot tracking algorithm to an adult’s (between 5 feet and 6 feet height) leg dimension and also based on the point cloud density obtained from six Intel RealSense D415 sensors. These parameters could be fine-tuned based on the person physical dimensions and point cloud density. This lower leg point cloud was divided into left and right leg points clouds. To segment a current point cloud frame, Euclidean clustering, average leg dimensions (calculated from the point cloud data), and past point cloud frames were used.

### A. Euclidean Clustering

Point cloud points were divided into clusters based on the Euclidean distances between points [29]. The clustering tolerance was 50 mm, which implies that the points within 50 mm radial distance from an interested point in the point cloud were clustered together.

**FIGURE 4.**
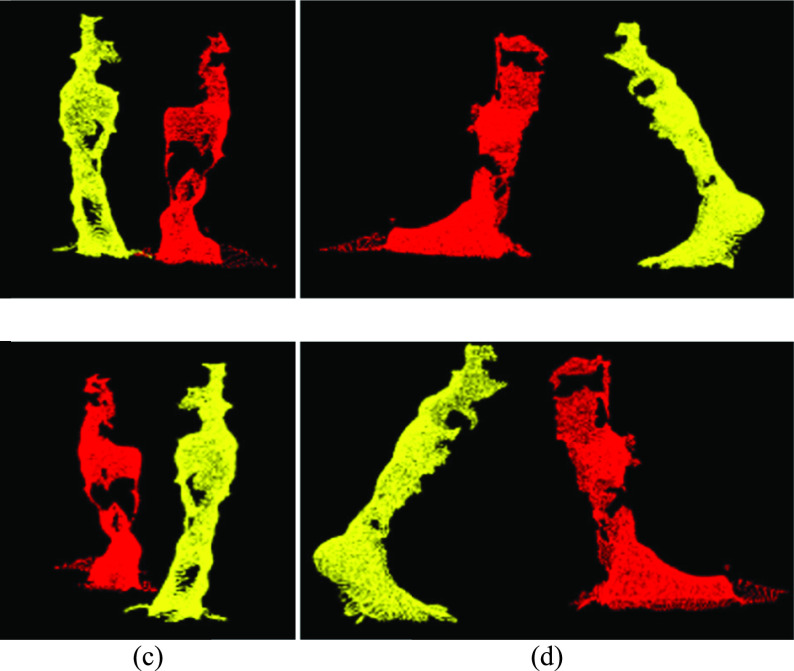
(a) Front, (b) left, (c) back, and (d) right views of the left (red) and right (yellow) leg points segmented using euclidean clustering.

Each cluster was verified using the number of points and cluster volume (i.e., volume of the bounding box around the cluster). Point clouds with two clusters, each with a minimum of 1000 points and cluster volume greater than 75 percent of the average leg volume were considered to contain data from two legs, and each cluster was considered an individual leg (Fig. 4). Point clouds with a single cluster greater than 1000 points and volume between 0.75 to 1.25 times the average volume was considered single leg data.

**FIGURE 5.**
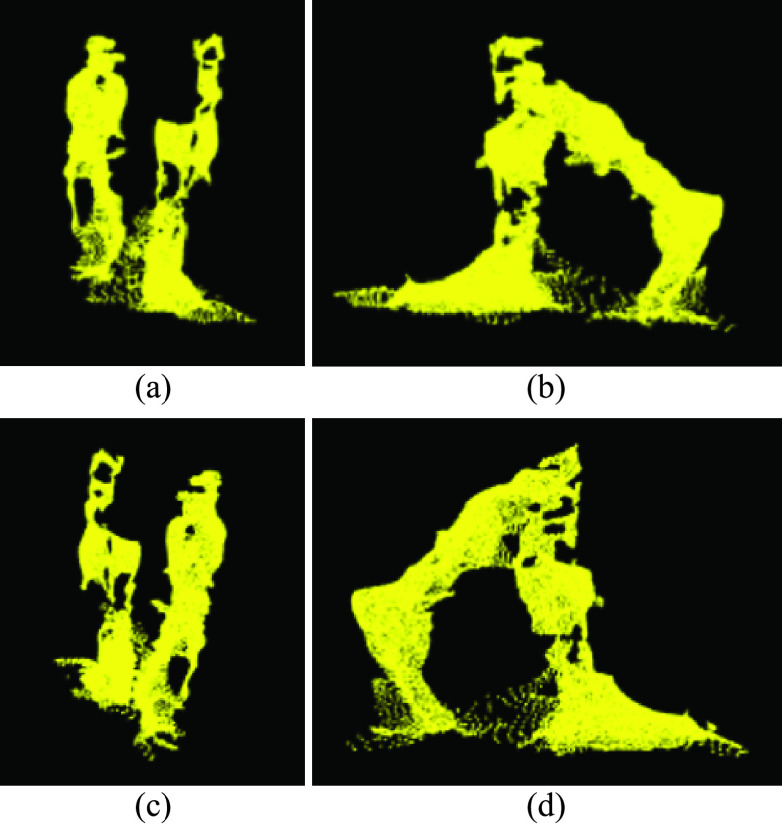
(a) Front, (b) left, (c) back, and (d) right views of a single clustered point cloud with two legs.

During mid-swing, when both legs are close together, noise between the legs caused the points to group into a single cluster. In these cases, the two legs data were identified as a single cluster (Fig. 5), with cluster volume greater than two times the average leg volume. Therefore, a different approach (“Moving points segmentation”) was used to segment the legs (Section IV-C).

Point cloud frames not in one of these three categories (Two valid legs, single leg, two legs as a single cluster) were ignored.

### B. Leg Dimensions

Leg dimensions were calculated from a closest oriented bounding box (OBB, Table 1) around the leg point cloud. Frames with two separate Euclidean clusters (two legs) and each leg with more than 1000 points were considered for calculating average leg dimensions, from 40 valid leg point clouds (Table 2). Dimensions ($l,w,h$) were calculated using Algorithm I (Table 1) and then sorted before calculating the average of the center 20 elements for each dimension.

**TABLE 1:** Algorithm I: Oriented Bounding Box Around Point Cloud Data

Step	Methodology
1	Calculate the point cloud’s ($PC$) centroid ($C$) and normalized covariance matrix (${COV}_{N}$). For ${COV}_{N}$, each covariance matrix element is divided by the number of points in the point cloud. The covariance matrix, also known as dispersion matrix, contains information on dispersion direction (Eigenvectors) and dispersion magnitude (Eigenvalues).
2	Calculate Eigenvectors ($\begin{aligned} \left [{ {\begin{array}{cccccccccccccccccccc} v_{1} & v_{2} & v_{3}\\ \end{array}} }\right] \end{aligned}$) for ${COV}_{N}$. Each vector is an OBB axis, where $v_{3} $ is replaced with a cross product of $v_{1}, v_{2}$ such that axes obey the right-hand rule.
3	Find an initial $4\times 4$ transformation matrix ($T_{OBB}^{init}$) formed using centroid and Eigenvectors as $\begin{aligned} T_{OBB}^{init}=\left [{ {\begin{array}{cccccccccccccccccccc} v_{1} & v_{2} & v_{3} & C\\ 0 & 0 & 0 & 1\\ \end{array}} }\right] \end{aligned}$
4	Find point cloud ${PC}_{o}$ with centroid at origin by transforming $PC$. The relation between point $p_{s}$ in $PC$, its corresponding point $p_{o}$ in ${PC}_{o}$ is $\begin{aligned} \left [{ {\begin{array}{cccccccccccccccccccc} p_{s}\\ 1\\ \end{array}} }\right]= T_{OBB}^{init}\left [{ {\begin{array}{cccccccccccccccccccc} p_{o}\\ 1\\ \end{array}} }\right] \end{aligned}$. A transformed ${PC}_{o}$ is shown in Fig. 7(a).
5	Find the minimum (${min}_{x}, {min}_{y}, {min}_{z}$) and maximum (${max}_{x}, {max}_{y}, {max}_{z}$) of each $x, y, z$ dimension. Obtain OBB dimensions from the absolute difference between minimum and maximum values in each dimension.
6	Calculate OBB position ($\begin{aligned} {POS}_{OBB}) \left [{ {\begin{array}{cccccccccccccccccccc} {POS}_{OB}\\ 1\\ \end{array}} }\right]=T_{OBB}^{init}\left [{ {\begin{array}{cccccccccccccccccccc} \frac {min_{x}+ {max}_{x}}{2}\\ \frac {min_{y}+ {max}_{y}}{2}\\ \frac {min_{z}+ {max}_{z}}{2}\\ 1\\ \end{array}} }\right] \end{aligned}$ The OBB rotation matrix is $\begin{aligned} R_{OBB}^{init}=\left [{ {\begin{array}{cccccccccccccccccccc} v_{1} & v_{2} & v_{3}\\ \end{array}} }\right]_{3 \times 3} \end{aligned}$. With these dimensions, position, and rotation, an OBB around the leg point cloud can be drawn (Fig. 7(b)).
7	Since rotations are not unique, ${PC}_{o}$ could be aligned in different ways (Fig. 8). The following steps ensure that foot AP is along the $x-axis$, ML is along the $y-axis$, and the bottom of the foot is in negative $z-axis$. This aligned point cloud at origin is represented as ${PC}_{o}^{eqnarray}$.
8	The four closest corners to floor are considered OBB bottom corners. The rest are top corners. Any bottom corner can be considered the reference point. Distances from the reference point to the remaining three bottom corners are calculated. Minimum distance is OBB width ($w$), maximum distance is diagonal, and middle distance is OBB length ($l$). The minimum distance from the reference corner to the top corners is the OBB height ($h$).
9	From a reference point, calculate a unit vector in the length direction ($\hat {l}$) and a unit vector in the height direction ($\hat {h}$).
10	Calculate the rotation matrix ($R_{OBB}$) between a coordinate system with point $O(0,0,0)$ as the origin, unit vectors $\hat {x}\left ({1,0,0 }\right)$, and $\hat {z}\left ({0,0,1 }\right)$ and the other coordinate system with corresponding values ${POS}_{OBB}, \hat {l}, $ and $\hat {h}$. This $R_{OBB}$ is the new rotation matrix for the OBB.

**TABLE 2:** Algorithm II: Valid Frames to Measure Dimensions

Step	Methodology
1	Calculate ${POS}_{OBB}, R_{OBB}, l, w, and h$ for a leg point cloud (${PC}_{l}$), using Algorithm I (Table 1).
2	For every point ($p_{l}$) in ${PC}_{l}$, a corresponding point ($p_{o}$) in the point cloud ${PC}_{o}^{eqnarray} $ is calculated using $\begin{aligned} \left [{ {\begin{array}{cccccccccccccccccccc} p_{o}\\ 1\\ \end{array}} }\right]= \left [{ {\begin{array}{cccccccccccccccccccc} R_{OBB} & {POS}_{OBB}\\ 0 & 1\\ \end{array}} }\right]^{-1}\left [{ {\begin{array}{cccccccccccccccccccc} p_{l}\\ 1\\ \end{array}} }\right] \end{aligned}$
3	A box with centroid at the origin, height ($h$) in the z-axis, width ($w$) in the y-axis, and length ($l$) in the x-axis bounds the ${PC}_{o}^{eqnarray}$.
4	Divide this box into 12 horizontal slices along the z-axis, starting from the bottom, with 25 mm height, length ($l$), and width ($w$).
5	For each slice, calculate minimum (${min}_{x}, {min}_{y}, {min}_{z}$) and maximum (${max}_{x}, {max}_{y}, {max}_{z}$) for each $x, y, z$ dimension using points inside the slice. Calculate the slice volume as $V_{slice}=({max}_{x}-{min}_{x})\times ({max}_{y}-{min}_{y})\times 25$.
6	A leg point cloud is considered valid for calculating dimensions if the maximum $V_{slice}$ was one of the bottom two slices and value greater than 80 percent of $l\times w \times 25$.

### C. Moving Points Segmentation

For a current point cloud frame (${PC}_{t}$) with two legs identified as a single cluster, a reference point cloud (${PC}_{ref}$) from the past frames was found using Algorithm III (Table 3).

**TABLE 3:** Algorithm III: Past Reference Point Cloud Frame

Step	Methodology
1	Project point cloud data (${PC}_{t}$) of the $N^{th}$ frame onto the floor plane and calculate centroid on the floor ($C_{N}$).
2	Reference frame at $R=N-5$ and $R > 0$.
3	If the number of points in the $R^{th}$ frame’s point cloud is less than 1000, go to step 6.
4	Project point cloud data at $R^{th}$ frame onto the floor plane and calculate centroid on the floor ($C_{R}$).
5	$R^{th}$ frame is the reference point cloud (${PC}_{ref}$), if the distance between $C_{N}$ and $C_{R}$ is greater than 100mm else go to the next step.
6	For $R=R-5$; if $R > 0$ go to step 3, else ignore the $N^{th}$ frame.

From every point in the reference point cloud (${PC}_{ref}$), points within 20 mm in ${PC}_{t}$ were categorized as the non-moving leg point cloud ${PC}_{1}$ (repeated points were ignored).

**FIGURE 6.**
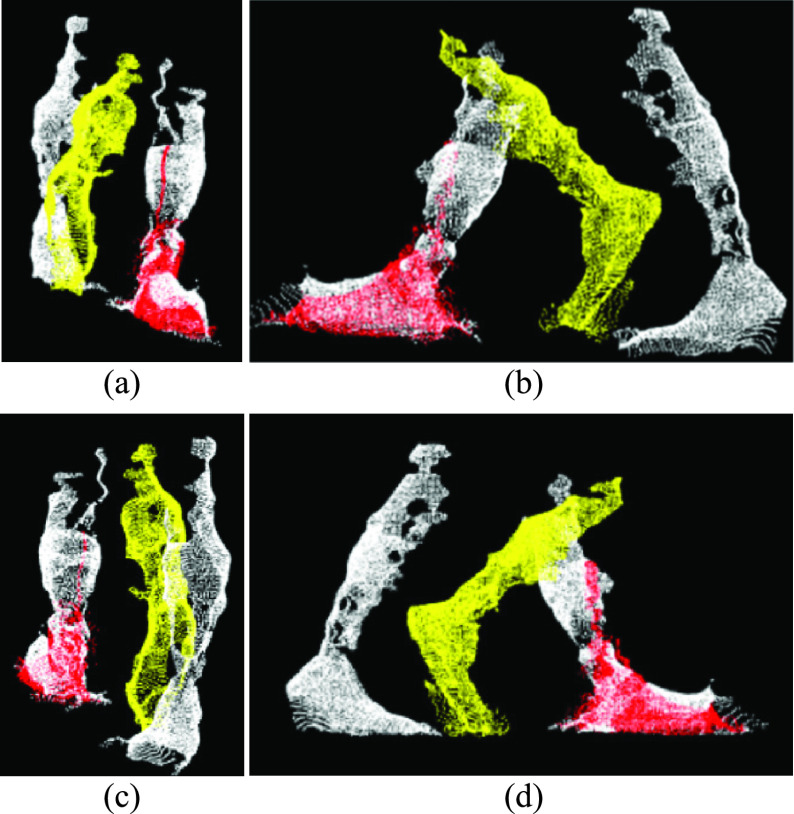
(a) Front, (b) left, (c) back, and (d) right views of a reference point cloud frame (white) and current point cloud frame segmented into non-moving leg points (red) and moving leg (yellow).

All other points in ${PC}_{t}$ were moving points and categorized as the other leg’s point cloud ${PC}_{2}$. Each point cloud had at least 30 percent of the total points in ${PC}_{t}$ and the statistical outlier removal filter was applied (Section III-D). Euclidean clustering (Section IV-A) was applied to ${PC}_{1}$ and ${PC}_{2}$, the biggest cluster from each point cloud was considered (Fig. 6).

**FIGURE 7.**
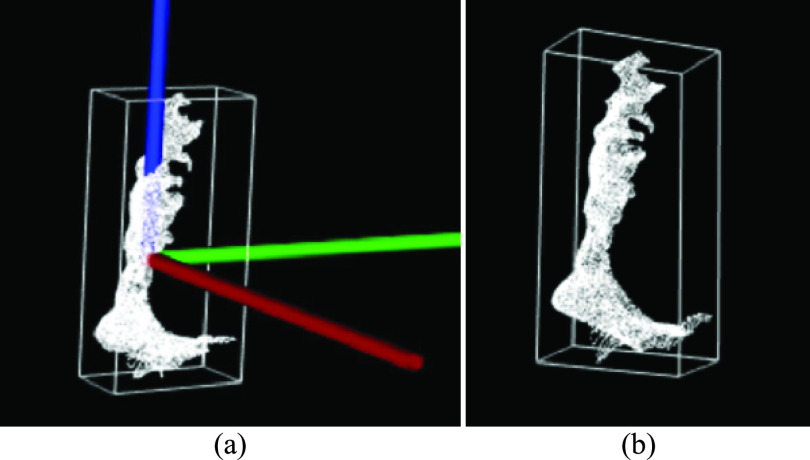
(a) Point cloud ${PC}_{o}$ with centroid at origin, (b) OBB around $PC$ (Red: $x-axis$, Green: $y-axis$, and Blue: $z-axis$).

**FIGURE 8.**
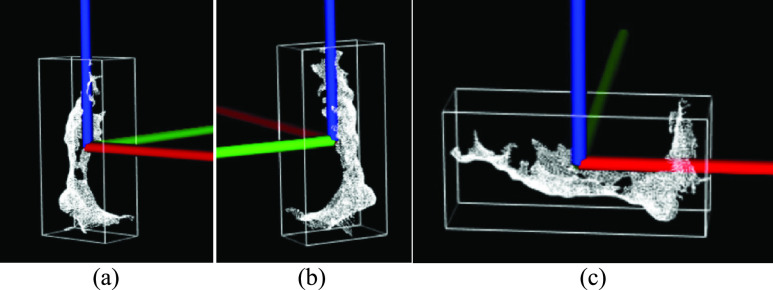
Leg ${PC}_{o}$ with AP of the foot along (a) $x-axis$ (red), (b) $y-axis$ (green), (c) $z-axis$ (Blue).

## V. Foot Tracking

Foot tracking was achieved from point cloud data by fitting a box with average foot dimensions around each foot, in each frame. The foot’s bottom plane was calculated and used to define bounding box rotation and position. The foot’s heel and toe points were based on the walking direction.

### A. Foot Dimensions

Using Algorithm II (Table 2), for a valid frame, the volume of points in 12 slices were calculated. Each slice’s volume was median filtered with both adjacent slice volumes using filter size = 3 and filter stride length = 1 (first and last elements were untouched).

The cut-off slice (i.e., slice defining top of foot) was defined by identifying the slice with the maximum volume ($V_{slice}^{max}$) and then scanning upwards to find the slice with volume less than 60 percent of $V_{slice}^{max}$. The points below this cut-off slice defined the foot. An OBB was calculated around these points (Table 1, Algorithm I) and OBB dimensions were foot length ($f_{l}), $ foot width ($f_{w}$), and foot height ($f_{h}$). These dimensions were found for 40 frames, values of each dimension were sorted and the center 20 elements were averaged.

### B. Foot Oriented Bounding Box

The foot’s bottom plane was found using Algorithm IV (Table 4). Points above this plane within the distance $f_{h}$ were considered to belong to foot point cloud (${PC}_{foot}$) and points between 0.1 times $f_{h}$ and 0.9 times $f_{h}$ were segmented as the center foot’s point cloud (${PC}_{foot}^{center}$).

**TABLE 4:** Algorithm IV: Foot Bottom Plane

Step	Methodology
1	Apply Algorithm I to a leg point cloud (${PC}_{L}$) and align the outputs at origin (${PC}_{o}^{eqnarray}$), OBB position (${POS}_{OBB}$), rotation ($R_{OBB}$), length ($l$), width ($w$), and height ($h$).
2	From an OBB bottom corner around ${PC}_{o}^{eqnarray}$, five sub-boxes with length ($l/4$), width ($w$), and height ($h/10$) are taken. The two outer bottom corner points of the first sub-box from the bottom, with a minimum of 50 points, are transformed using ${PO??}_{OBB}$ and $R_{OBB}$. These transformed points ($p_{c}^{OBB}$) are the foot OBB corners.
3	Repeat step 2 for the foot’s other side.
4	From a point $p_{c}^{OBB}$, the centroid of thirty closest points in ${PC}_{L}$ is considered the foot bottom point ($f_{b}$). Repeat to find all the four bottom points on the foot.
5	Using these four $f_{b}$ points, four plane equations are obtained by taking different combinations of three points and distance from the fourth point to the plane is calculated. The plane with the smallest distance from the fourth point is the “Best Plane”.
6	Points within 5 mm above the best plane are fitted to a RANSAC plane model [30] and the output considered as foot’s bottom plane.

${PC}_{foot}^{center}$ points were projected onto the foot’s bottom plane and then Algorithm I was partially applied (until step 6). OBB rotation around the foot (${R_{OBB}^{foot}= R}_{OBB}^{init}$) was obtained with dimensions ($x_{OBB}^{foot},y_{OBB}^{foot},z_{OBB}^{foot}$). Then OBB dimensions mapped with average foot dimensions as the minimum of $x_{OBB}^{foot}, y_{OBB}^{foot},z_{OBB}^{foot}$ were replaced with $f_{h}$, maximum with $f_{l}$, and the remaining dimension with $f_{w}$.

The foot’s OBB position (${POS}_{OBB}^{foot}$) was the centroid of ${PC}_{foot}$. Position (${POS}_{OBB}^{foot}$), rotation ($R_{OBB}^{foot}$), and dimensions ($x_{OBB}^{foot}\mathrm {, }y_{OBB}^{foot},z_{OBB}^{foot}$) were used to locate a box around the foot.

### C. Heel and Toe Segmentation

The point cloud data was transformed using 5000 mm translations in the $x$ and $y$ axes such that the walking pathway was always in the positive *xy*-plane. This reduced the complexity of further processing and understanding.

Left and right leg segmentation was based on the walking direction, calculated using OBB centroid trajectory. When walking towards the origin along a pathway parallel to the $y$-axis, the leg closer to the $y$-axis was the right leg and the other leg was labelled as left. Opposite leg classification was applied when walking away from the origin.

**FIGURE 9.**
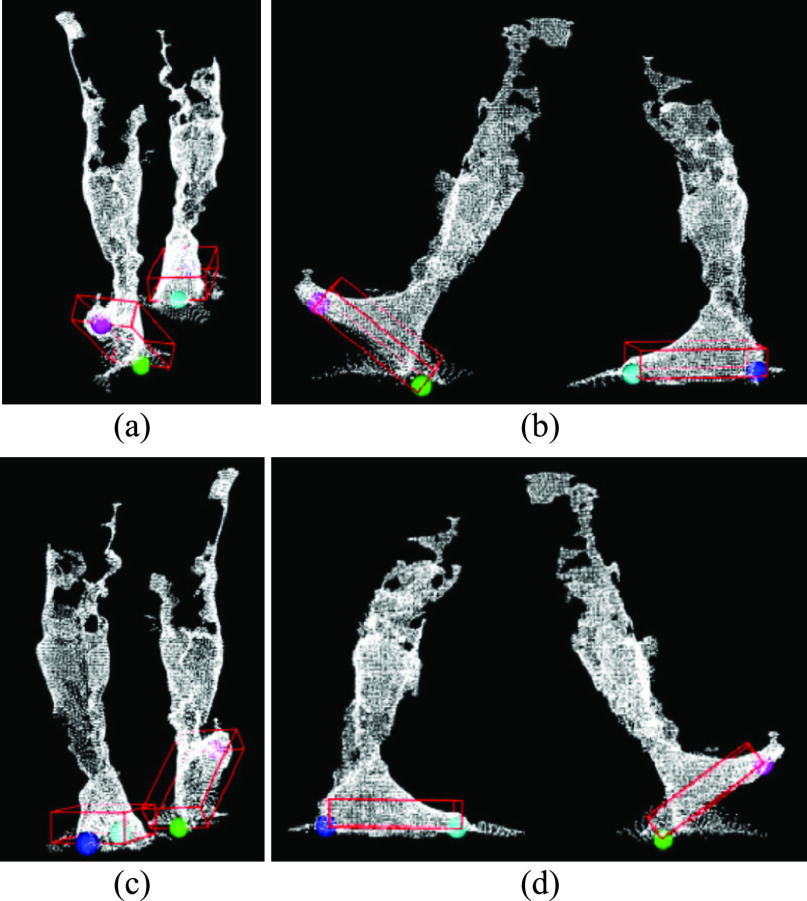
(a) Front, (b) left, (c) back, and (d) right views of a point cloud frame with heel and toe segmentation (right heel – green, right toe – magenta, left heel – blue, left toe – cyan).

For each foot OBB, the center point ($p_{OBB}^{toe}$) of the front two bottom corners and the center point ($p_{OBB}^{heel}$) of the back two bottom corners were calculated using Algorithm V (Table 5). These points were considered as the toe and heel, respectively (Fig. 9).

**TABLE 5:** Algorithm V: Heel and Toe Segmentation

Step	Methodology
1	Calculate OBB centroid ($c_{x}^{OBB}, c_{y}^{OBB}, c_{z}^{OBB}$).
2	Identify OBB four front corners and four back corners. $\begin{aligned} \mathrm {front corner}{\begin{cases} \mathrm {y-axis value < }c_{y}^{OBB}\mathrm {;walking towards} \\ \mathrm {y-axis value > }c_{y}^{OBB}\mathrm {;walking away} \\ \end{cases}}\,\,\mathrm {back corner}{\begin{cases} \mathrm {y-axis value > }c_{y}^{OBB}\mathrm {;walking towards} \\ \mathrm {y-axis value < }c_{y}^{OBB}\mathrm {;walking away} \\ \end{cases}} \end{aligned}$
3	Calculate centroid of front corners (${fc}_{x}^{OBB}, {fc}_{y}^{OBB}, {fc}_{z}^{OBB}$) and back corners (${bc}_{x}^{OBB}, {bc}_{y}^{OBB}, {bc}_{z}^{OBB}$).
4	From front corners, front left and front right corners are: $\begin{aligned} \mathrm {front left}{\begin{cases} \mathrm {x-axis value > }{fc}_{x}^{OBB}\mathrm {;walking towards} \\ \mathrm {x-axis value < }fc_{x}^{OBB}\mathrm {;walking away} \\ \end{cases}}\,\,\mathrm {front right}{\begin{cases} \mathrm {x-axis value < }{fc}_{x}^{OBB}\mathrm {;walking towards} \\ \mathrm {x-axis value > }{fc}_{x}^{OBB}\mathrm {;walking away} \\ \end{cases}} \end{aligned}$
5	From back corners, back left and back right corners are: $\begin{aligned} \mathrm {back left}{\begin{cases} \mathrm {x-axis value > }{bc}_{x}^{OBB}\mathrm {;walking towards} \\ \mathrm {x-axis value < }bc_{x}^{OBB}\mathrm {;walking away} \\ \end{cases}}\,\,\mathrm {back right}{\begin{cases} \mathrm {x-axis value < }{bc}_{x}^{OBB}\mathrm {;walking towards} \\ \mathrm {x-axis value > }{bc}_{x}^{OBB}\mathrm {;walking away} \\ \end{cases}} \end{aligned}$
6	For front, the front left bottom corner has a lower z-axis value and the other is the front left top corner. The same logic is applied to front right corners, back left corners, and back right corners.
7	Calculate foot length, width, and height and validated with average foot dimensions ($f_{l}, f_{w},f_{h}$). $\begin{aligned} \mathrm {foot length=}{\begin{cases} \left \|{ \mathrm {front left bottom-back left bottom} }\right \| \\ \left \|{ \mathrm {front left top-back left bottom} }\right \| \\ \end{cases}}\,\,\mathrm {foot width=}{\begin{cases} \left \|{ \mathrm {front left bottom-front right bottom} }\right \| \\ \left \|{ \mathrm {front left top-front right top} }\right \| \\ \end{cases}}\,\,\mathrm {foot height=}{\begin{cases} \left \|{ \mathrm {front right bottom-front right top} }\right \| \\ \left \|{ \mathrm {back left bottom-back left top} }\right \| \\ \end{cases}} \end{aligned}$
8	OBB toe point is the center of front bottom two points ($p_{OBB}^{toe}$) and heel point is the center of back bottom two points ($p_{OBB}^{heel}$).

## VI. Validation

The foot tracking algorithm was validated by comparing gold standard Vicon system output with the marker-less smart hallway system. Volunteer walking trials were captured simultaneously with both the systems and post-processing filters were applied. This section describes the data collection protocol and post-processing processes.

### A. Protocol

**FIGURE 10.**
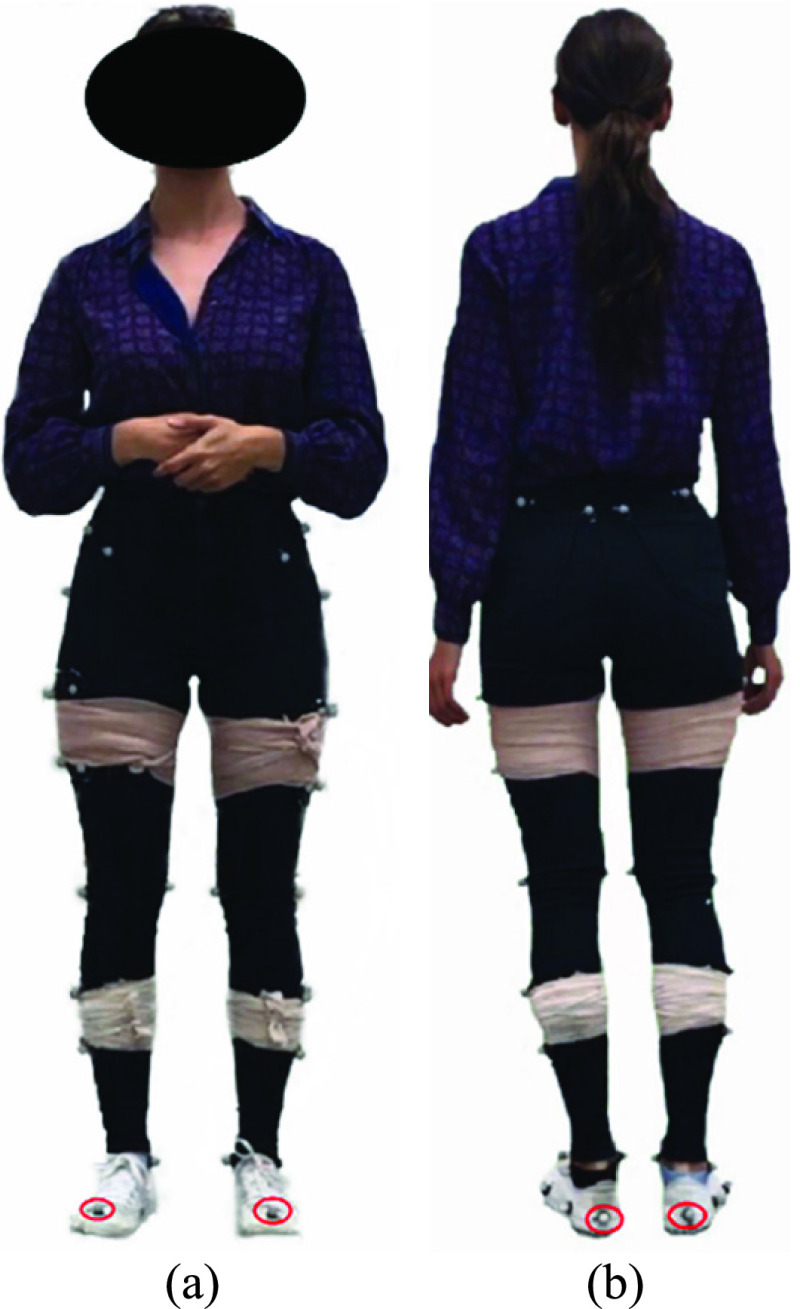
(a) Front and (b) back view of a participant with reflective markers.

Twenty-two able-bodied volunteers were recruited from students and staff at the University of Ottawa. After informed consent, reflective markers were attached to the participant’s lower body (Fig. 10) (foot markers were used in this application) and then the participant walked 12 times with their natural gait and comfortable speed along a walkway with a 1.5m capture zone. Data were captured simultaneously with a 13 camera Vicon system at 100 Hz [31] and the new marker-less system at approximately 60 Hz. Since the Vicon system captured more than the 1.5m walkway, the data within the capture zone of both systems were used for calculating the stride parameters. This protocol was approved by the Research Ethics Board of the University of Ottawa (File number: H-08-18-860, Approval date: 29-10-2018) [32].

In this study, Vicon and the new marker-less system were not synchronized in time. Even though both systems captured data simultaneously, each system was independent. Stride parameters were calculated individually, then synchronized based on spatial foot events information.

### B. Post-Processing

3D positions of left toe, left heel, right toe, and right heel markers were reconstructed using Vicon Nexus software [33]. Gaps in the trial data were filled using cubic spline interpolation and then filtered using a 4th order dual-pass Butterworth lowpass filter with 20 Hz cut-off frequency.

Marker-less point cloud data were constructed from the depth images, then 3D locations of toes and heels were tracked. Left toe, left heel, right toe, and right heel were processed independently. Data outliers were statistically filtered, with values two standard deviations or more from the mean removed. Based on time stamp information, trajectory gaps were filled using cubic spline interpolation.

Since the capture time between frames was inconsistent, cubic spline interpolation was used to re-sample the data to 60Hz. This re-sampled data was low pass filtered using 4th order dual-pass Butterworth filter with a cut-off frequency of 12Hz. Using cubic spline interpolation, the low pass filtered data was then re-sampled gain to the originally captured time stamps.

## VII. Stride Parameters

This section describes the stride parameters used with both Vicon and marker less systems. The stride parameters were calculated by finding the foot events from the segmented heel and toe points obtained from the foot-tracking algorithm.

Foot events such as foot strike (FS) and foot-off (FO) were identified to calculate stride parameters. Vertical foot coordinates ($z$-axis) were used to identify FS and FO frames [34].

### A. Foot Events

#### 1) VICON

**FIGURE 11.**
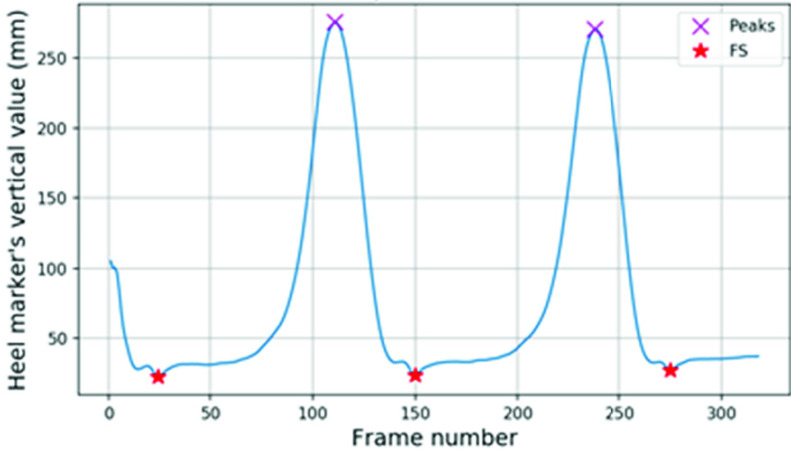
FS events from the Vicon heel marker’s vertical values.

**FIGURE 12.**
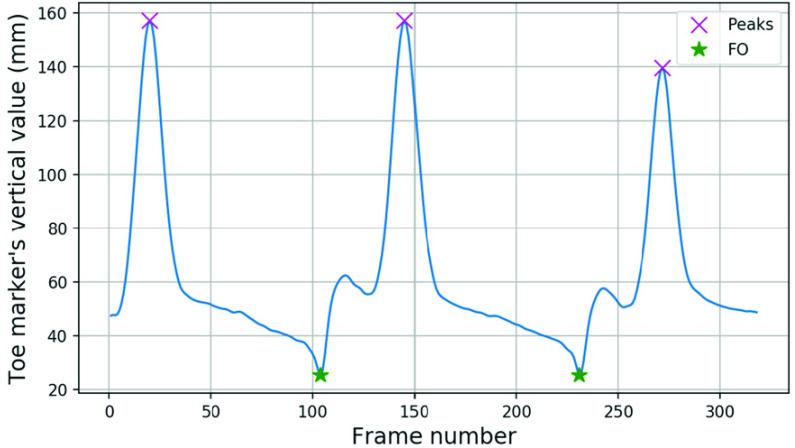
FO events from the Vicon toe marker’s vertical values.

Peak vertical values in swing phase were detected for heel (Fig. 11) and toe (Fig. 12) markers. These peaks were based on the zero cross over from positive to negative in the vertical velocity, then a peak value greater than 75 percent of the maximum vertical value condition was applied.

Between two peaks, FS and FO should only occur once. Zero crossovers from negative to positive in the vertical velocity were concave shaped dips in the vertical displacement graph. These concave dips within the bottom 20 percent of the vertical range were identified. The FS frame was the minimum dip between the two peaks in heel data (Fig. 11) and FO was the minimum dip in toe data (Fig. 12).

Additional conditions were applied to the minimum concave dips before the first peak and after the last peak. The minimum concave dip before the first peak with a distance (in frames) less than 50 percent of the frame length between the first two heel peaks was ignored, and greater than 50 percent in the toe data was ignored. Similarly, the number of frames between the last peak and the minimum concave dip after the last peak must be less than 50 percent of the frame length between the last two peaks for heel data and greater than 50 percent for toe data.

#### 2) Marker-Less

Vertical direction data from the Marker-less system was not as smooth as the Vicon data. The foot event frames were initially estimated using AP (y-axis) data, then finalized based on the vertical data.

**FIGURE 13.**
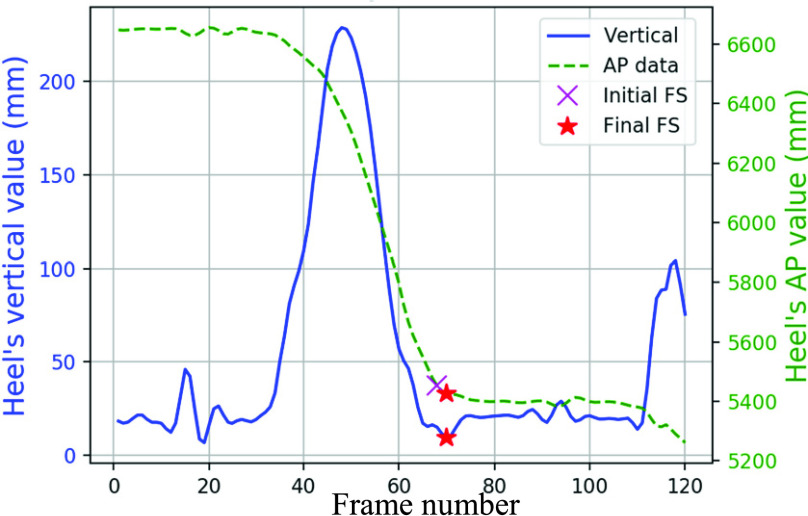
FS events in marker-less system’s heel data.

**FIGURE 14.**
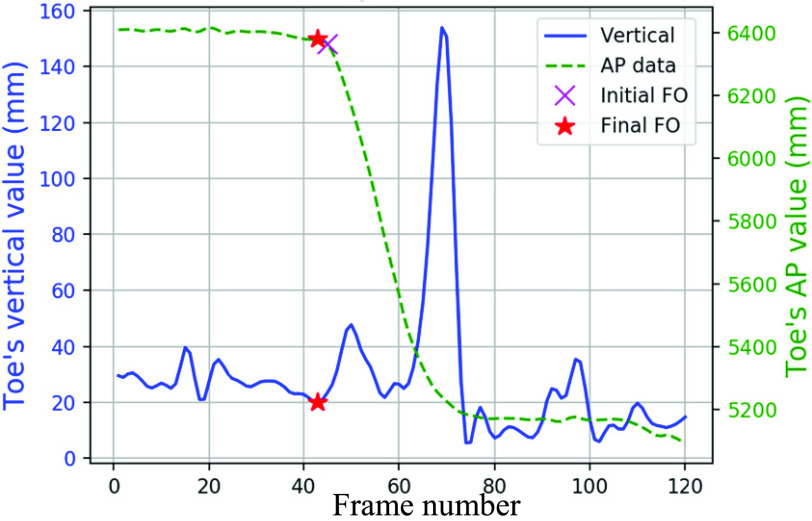
FO events in marker-less system’s toe data.

The frame where the foot reached a stationary state in the AP direction was considered the initial FS frame (Fig. 13). Vertical movements may occur after AP movements halted, so the closest concave dip within the next five frames in vertical data was considered as the final FS (Fig. 13). For cases with no concave dip, the initially estimated frame was considered the final FS. The final FO frame was determined from an initially estimated FO frame, where AP displacement began (Fig. 14), and five frames before the initial estimated FO frame in the vertical direction (Fig. 14). This method is detailed in Algorithm VI (Table 6).

**TABLE 6:** Algorithm VI: Foot Events Identification in the Marker-Less Data

Step	Methodology
1	Create a binary version of arrays for $AP$ heel data ($B_{AP}^{heel}$) and $AP$ toe data ($B_{AP}^{toe}$). For $AP$ data ($D$) with $N$ frames, the $i^{th}$ frame in binary version ($B$) is calculated as $\begin{aligned} B\left ({i }\right)={\begin{cases} 1, \& \left |{ D\left ({i }\right)-D(i+1) }\right | > 20 and i < N \\ 1, \& \left |{ D\left ({i }\right)-D(i-1) }\right | > 20 and i=N \\ 0, \& else \\ \end{cases}} \end{aligned}$
2	Median filter binary arrays with filter size = 3 and filter stride = 1.
3	In the sequence of binary heel frames, the transition frame from high to low with at least three high-valued frames and two low-valued frames is the initial FS frame.
4	The closest zero cross-over frame from negative to positive in the heel’s vertical velocity within the next five frames after the initial FS frame is the final FS frame. If no such frame exists, then initial and final FS frame are the same.
5	In the sequence of binary toe frames, the transition frame from low to high with at least three low-valued frames and two high-valued frames is the initial FO frame.
6	The closest zero cross over frame from negative to positive in the toe’s vertical velocity within the before five frames of the initial FO frame is the final FO frame. If no such frame exists, then the initial and final FS frame are the same.

Left Foot Strike (LFS), Left Foot-Off (LFO), Right Foot Strike (RFS), and Right Foot-off (RFO) were validated according to the normal gait cycle event sequence (i.e., after LFO, the expected next event is LFS and then RFO). If multiple LFS events are identified before RFO, the closest to the RFO event was considered. If no LFS event was identified between LFO and RFO, then the events were ignored.

### B. Results

The stride parameters in this research were from one gait cycle (Table 7). Primary parameters were directly obtained from the tracking data and the derived parameters were calculated from the primary parameters.

**TABLE 7:** Stride Parameters Calculations in a Normal Gait Cycle

Parameter	Definition
Left stride length	Distance covered by the left heel between two consecutive LFS along the sagittal plane (line of progression, $y-axis$)
Right stride length	Sagittal distance covered by the right heel between two consecutive RFS
Left stride time	Time between two consecutive LFS
Right stride time	Time between two consecutive RFS
Left stride speed^d^	Left stride length / Left stride time
Right stride speed^d^	Right stride length / Right stride time
Left step length	Sagittal distance between right and left heels at LFS
Left step width	Frontal distance ($x-axis$) between right and left heels at LFS
Left step time	Time between RFS and LFS
Right step length	Sagittal distance between left and right heels at RFS
Right step width	Frontal distance between left and right heels at RFS
Right step time	Time between LFS and RFS
Left cadence^d^	Left steps per minute
Right cadence^d^	Right steps per minute
Left stance time	Time between LFS and LFO
Left swing time	Time between LFO and LFS
Left stance to swing ratio^d^	Left stance time / Left swing time
Right stance to swing ratio^d^	Right stance time / Right swing time
Left double support time	Time between LFS and RFO
Right double support time	Time between RFS and LFO
Left foot angle	Angle of left foot midline with sagittal plane when the left foot is entirely in contact with the floor.
Right foot angle	Angle of right foot midline with sagittal when the right foot is entirely in contact with the floor.
Left foot maximum velocity	Maximum left foot velocity during the left swing phase (LFO to LFS)
Right foot maximum velocity	Maximum right foot velocity during the right swing phase (RFO to RFS)
Left foot clearance	Minimum vertical distance of left foot during mid-swing
Right foot clearance	Minimum vertical distance of right foot during its mid-swing

^d^ Derived stride parameters

Foot events from Vicon and marker-less systems were synced based on foot position of the first common foot event in the marker-less system.

Stride parameters from the both systems were compared and analyzed. For $n$ samples, with $i^{th}$ sample represented as $x_{i}$, the mean ($\mu$) and standard deviation ($\sigma$) were calculated using (9) and (10), respectively. For a parameter, with value $v$ from the Vicon system and value $m$ from marker-less system, the sample error ($e$) was calculated using (11).

For each primary stride parameter, $\mu $ and $\sigma $ of the error values were calculated. Most values farther than two $\sigma \mathrm { }$ from the $\mu $ were due to false detection of foot events because of insufficient capturing volume and noisy data. Since these erroneous data was not because of improper foot tracking, it was categorized as outliers and removed from the analysis. Primary stride parameter inliers were used to calculate derived stride parameters. For a stride parameter with $N$ samples (inliers), $i^{th}$ Vicon’s sample $v_{i}$, $i^{th}$ marker-less system’s sample $m_{i}$, the mean error ($e_{\mu }$), error’s standard deviation ($e_{\sigma }$), absolute mean error ($e_{\mu }^{abs}$), absolute error’s standard deviation ($e_{\sigma }^{abs}$), minimum error ($e_{min}$), maximum error ($e_{max}$), Pearson coefficient ($r$), and the percentage of inliers ($I_{\%}$) were calculated (Table 8).

**TABLE 8:** Marker-Less Stride Analysis

Parameter (units)		Vicon ($\mu \pm \sigma$)	Marker-less ($\mu \pm \sigma$)	Error ($e_{\mu } \pm e_{\sigma }$)	$e_{\mu }^{abs}\pm e_{\sigma }^{abs}$	$e_{min}$	$e_{max}$	$r$	Inliers count	$I_{\%}$
Left	Stride speed (m/s)	1.06 ± 0.11	1.05 ± 0.11	0.012 ± 0.02	0.018 ± 0.02	−0.03	0.07	0.98	–	–
	Stride length (m)	1.29 ± 0.09	1.28 ± 0.09	0.012 ± 0.03	0.023 ± 0.02	−0.05	0.12	0.95	90	97.8
	Stride time (s)	1.22 ± 0.09	1.23 ± 0.1	−0.002 ± 0.04	0.027 ± 0.02	−0.10	0.11	0.93	87	94.5
	Cadence (steps/min)	100.95 ± 8.5	100.79 ± 9.29	0.159 ± 5.35	4.043 ± 3.51	−22.22	15.48	0.82	–	–
	Step length (cm)	66.32 ± 5.14	66.07 ± 5.52	0.247 ± 2.45	1.825 ± 1.66	−13.46	8.93	0.90	207	92.0
	Step width (cm)	9.65 ± 4.06	7.24 ± 4.4	2.415 ± 2.64	2.986 ± 1.97	−5.28	8.74	0.81	213	94.6
	Step time (s)	0.6 ± 0.05	0.60 ± 0.06	−0.002 ± 0.03	0.024 ± 0.02	−0.10	0.10	0.85	221	98.2
	Stance time (s)	0.78 ± 0.07	0.79 ± 0.08	−0.008 ± 0.03	0.024 ± 0.02	−0.10	0.07	0.92	185	97.8
	Swing time (s)	0.42 ± 0.04	0.43 ± 0.04	−0.003 ± 0.03	0.028 ± 0.02	−0.08	0.08	0.60	173	96.6
	Stance to swing ratio (NA)	1.86 ± 0.14	1.90 ± 0.22	−0.031 ± 0.21	0.172 ± 0.12	−0.67	0.38	0.41	–	–
	Double support time (s)	0.17 ± 0.03	0.18 ± 0.04	−0.004 ± 0.03	0.027 ± 0.02	−0.11	0.10	0.53	248	98.4
	Foot angle (°)	8.34 ± 5.14	1.12 ± 0.63	7.209 ± 5.13	7.256 ± 5.06	−1.22	19.14	0.08	176	93.6
	Foot max. velocity(m/s)	3.71 ± 0.31	3.88 ± 0.37	−0.163 ± 0.28	0.247 ± 0.22	−1.01	0.45	0.66	171	95.5
	Foot clearance (cm)	2.6 ± 0.62	1.99 ± 1.36	0.605 ± 1.39	1.224 ± 0.90	−4.19	3.07	0.18	113	93.4
Right	Stride speed (m/s)	1.1 ± 0.12	1.09 ± 0.12	0.006 ± 0.02	0.017 ± 0.01	0.06	−0.04	0.98	–	–
	Stride length (m)	1.29 ± 0.10	1.29 ± 0.10	0.001 ± 0.02	0.017 ± 0.01	0.06	−0.04	0.97	79	96.3
	Stride time (s)	1.19 ± 0.09	1.19 ± 0.09	−0.007 ± 0.03	0.025 ± 0.02	0.08	−0.07	0.95	77	93.9
	Cadence (steps/min)	99.44 ± 7.55	99.28 ± 7.95	0.157 ± 4.68	3.678 ± 2.89	12.7	−12.72	0.82	–	–
	Step length (cm)	64.80 ± 4.96	64.42 ± 5.41	0.3767 ± 2.19	1.722 ± 1.40	8.25	−8.82	0.91	177	89.8
	Step width (cm)	9.54 ± 3.76	6.80 ± 4.30	2.742 ± 2.75	3.227 ± 2.16	8.86	−5.19	0.77	185	93.9
	Step time (s)	0.61 ± 0.05	0.61 ± 0.05	−0.001 ± 0.03	0.023 ± 0.02	0.10	−0.08	0.82	193	97.9
	Stance time. (s)	0.77 ± 0.07	0.78 ± 0.08	−0.009 ± 0.03	0.025 ± 0.02	0.08	−0.10	0.92	206	98.5
	Swing time. (s)	0.43 ± 0.03	0.42 ± 0.04	0.003 ± 0.03	0.023 ± 0.02	0.08	−0.06	0.69	141	94.6
	Stance to swing (NA)	1.82 ± 0.13	1.85 ± 0.18	−0.027 ± 0.15	0.127 ± 0.09	0.38	−0.36	0.56	–	–
	Double support (s)	0.17 ± 0.03	0.17 ± 0.05	−0.002 ± 0.04	0.027 ± 0.02	0.10	−0.16	0.65	245	99.6
	Foot angle (°)	9.05 ± 5.71	1.14 ± 0.58	7.915 ± 5.62	7.935 ± 5.60	21.21	−0.92	0.20	201	96.2
	Foot max. velocity (m/s)	3.67 ± 0.28	3.83 ± 0.36	−0.155 ± 0.25	0.204 ± 0.21	0.32	−1.07	0.73	139	93.3
	Foot clearance (cm)	2.68 ± 0.62	2.10 ± 1.44	0.572 ± 1.35	1.251 ± 0.77	3.26	−3.35	0.36	102	96.2

The mean and absolute errors for step length and step time were within the minimum detectable change (MDC) for older people (age; mean = 78.09, standard deviation = 6.2) (step length MDC_95_ = 47 mm, step time MDC_95_ = 42 ms). However, step width was slightly greater than MDC (left step width mean error = 24.15 mm, left step width absolute error = 29.86 mm, right step width mean error = 27.42 mm, right step width absolute mean error = 32.27 mm, step width for older people MDC_95_ = 20 mm) [35]. The best Pearson correlation coefficient was for stride speed ($r=0.98$) and lower values were obtained for left foot angle ($r=0.08$) and left foot clearance ($r=0.18$).

### C. Discussion

The novel smart hallway system successfully tracked the foot and provided viable stride parameter output that could be used for decision-making, in most cases. The marker-less system had small mean absolute errors for the majority of stride parameters, compared with the Vicon system. For all the parameters, greater than 90% were inliers. Most outliers were due to limitations of capturing zone and noise from the sensors.

To the best of our knowledge, this research is the first to report foot clearance with marker-less depth sensors. A maximum absolute mean error of 1.25 cm was observed for right foot clearance, which was too large for clinical assessment purposes. With a marker-less system frame rate at approximately 60fps, all temporal stride parameters were accurate within 10 ms mean error and 30 ms absolute mean error. Errors in spatial stride parameters were due to “floor-plane to foot plantar surface” noise generated in the depth images during foot contact phases.

In comparison to the Kinect V2 based studies [16], [17], while mean errors of walking speed, stride length, and step length parameters were in similar range, step width mean errors were high, and temporal parameters’ mean errors (step time and stride time) showed better accuracy.

The new foot tracking algorithm, based on the fixed size OBB and foot bottom plane to define foot orientation, counteracted the AP noise to some extent. Average errors were higher in ML dependent stride parameters such as step width and foot angle.

Based on the errors and comparison with the MDC for older people [35], this novel marker-less system has potential to perform stride analysis on large population of older people in institutional hallways. This novel foot tracking algorithm could obtain more accurate stride parameters with better (less noisy) point cloud data.

\begin{align*}\mu=&\frac {1}{n}\sum \limits _{i=1}^{n} x_{i} \tag{9}\\ \sigma=&\sqrt [{2}]{\frac {\sum \limits _{i=1}^{n} {(x_{i}-\mu)}^{2}}{n}} \tag{10}\\ e=&v-m \tag{11}\\ e _{\mu }=&\frac {1}{N}\sum \limits _{i=1}^{N} {v_{i}- m_{i}} \tag{12}\\ e _{\sigma }=&\sqrt [{2}]{\frac {\sum \limits _{i=1}^{N} {(v_{i}-m_{i}-e_{\mu })}^{2}}{N}} \tag{13}\\ e _{\mu }^{abs}=&\frac {1}{N}\sum \limits _{i=1}^{N} \left |{ v_{i}-m_{i} }\right | \tag{14}\\ e _{\sigma }^{abs}=&\sqrt [{2}]{\frac {\sum \limits _{i=1}^{N} {(\left |{ v_{i}-m_{i} }\right |-e_{\mu }^{abs})}^{2}}{N}} \tag{15}\\ I _{\%}=&\frac {inliers ~count}{inliers~ count+outliers ~count} \times 100\tag{16}\end{align*}

## VIII. Conclusion

In this research, we proposed a smart hallway using depth sensors for foot tracking and stride parameter analysis. With six temporally and spatially synchronized Intel RealSense D415 depth sensors, depth data were successfully background-subtracted and merged to form a walking human’s point cloud time series. The point cloud was then effectively segmented into left and right foot point clouds. A bounding box was fitted around the foot in each leg’s point cloud data. The bounding box around the foot in each frame enabled foot tracking, and stride parameter calculation. Most stride parameters obtained from this newly developed marker-less system comparable favorably with gold standard Vicon system output.

While the marker-less system had promising results with accurate temporal stride parameters and small errors in spatial stride parameters, step width accuracy needs to improve and poor foot angle accuracy was observed due to noise around the foot as it approached the floor plane. Since foot clearance error was greater than 1 cm, and foot clearance varying between 2 and 3.2 cm, this error would need to be reduced to provide usable results for clinical decision-making.

Unlike the machine learning based skeleton tracking systems, foot landmarks from our proposed system never move outside the foot and data are captured at approximately 60 fps. This system could monitor a large number of people for long hours with no preparation time (no sensors attached to the body), without any discomfort, and without expert intervention.
